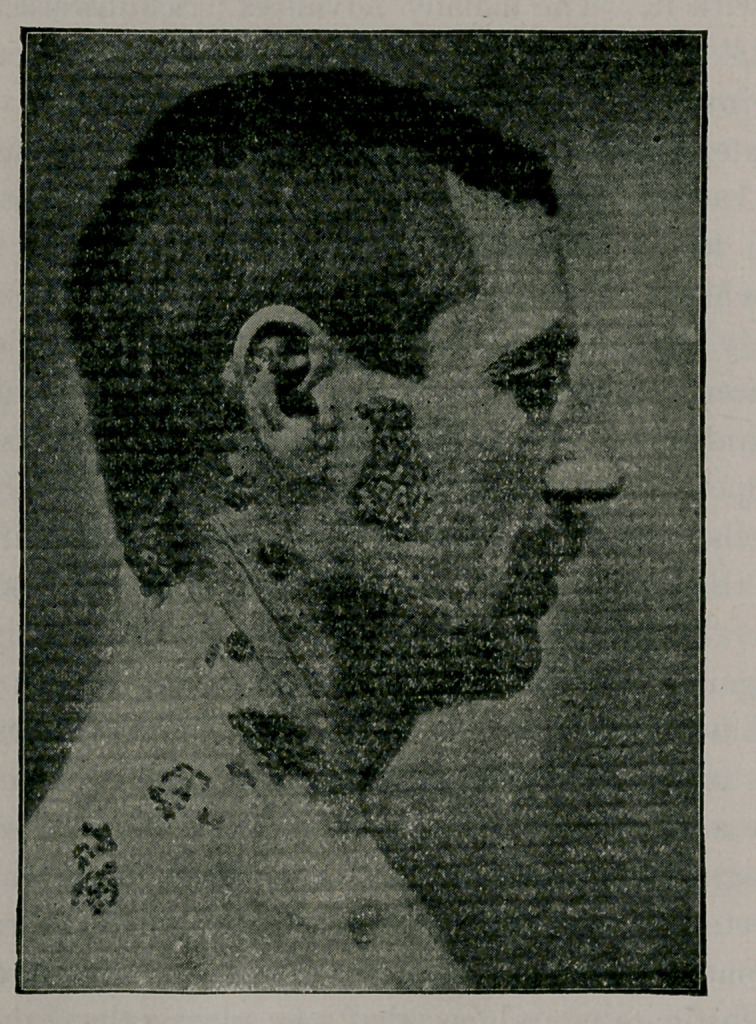# Zoster*A clinical lecture.

**Published:** 1895-07

**Authors:** Wm. S. Gottheil

**Affiliations:** Dermatologist to the Lebanon Hospital, and the Northwestern and the German West Side Dispensaries, New York


### ZOSTER.*

BY WM. S. GOTTHEIL, M.D.,

Dermatologist to the Lebanon Hospital, and the Northwestern and the German West Side Dispensaries, New York.

Several diseases have enjoyed the distinction of being sacred in the ancient popular nosologies; of being more directly than ordinary maladies the especial result of supernatural retribution. Leprosy was the physical scourge of the Deity among the ancient Jews, as witness the punishment of Naaman and others. The divine origin of epilepsy was believed in for long ages and in the most varied countries. Chorea was the dance of St. Vitus. In all the countries of the East insanity still marks the sufferer as the object of supernatural solicitude. Dermatology can show a similar example in the "Ignis Sacer" or "Sacred Fire," or "Fire of St. Anthony," more commonly known as "Herpes Zoster."

Zoster is derived from zona, meaning a ring or girdle, and herpes zoster means herpes in the form of a ring; of which designation the German "gurtel-rose," or "ring-rose," and "feuer-rose," or "fire-rose" are only popular synonyms. The English "shingles" is evidently a corruption of "cingula" or "cingulum," meaning the same

*A clinical lecture.

thing. Of late years the simple term "zoster" or "zona" has replaced the older herpetic designations.

Zoster is a moderately rare disease. My own statistics show thirty-two cases in eight thousand consecutive cases of skin disease in public and private practice. This is about the figure given by McCall Anderson and others. Every year several cases appear at our clinic. It is classed as a dermato-neurosis; but more exact examination shows it to be a symptomatic disease, a dystrophy in consequence of central or peripheral nerve anomalies of organic or functional character. Its evident connection, shown both by its distribution and by its general symptomatology, with the nerves, led Baerensprung in 1861 to refer it to an inflammatory affection of the spinal nerves. The opportunities for post-mortem examination in an affection that is self-limited, and but very rarely directly causes death, are of course but scanty; nevertheless, a sufficient number of cases in which complications or other diseases have caused a fatal termination are now on record to clarify our ideas of its pathology. There is inflammation of the tissues of the ganglia of the spinal or cerebral nerves. Inflammatory or hemorrhagic foci are found in these structures; and the various changes incident to neuritis occur in the nerve trunks leading from them. A number of cases have been reported in connection with traumatisms, compression by tumors, pachy-meningeal exudations, spinal caries, etc., in all of which the neuritis occurred as the primary phenomenon. The fact that the disease is more frequently seen at certain periods of the year than at others has led Wasielewski to claim that it is an infectious disease; further evidence in that direction being afforded by the immunity from subsequent recurrence which one attack seems to confer. This may be partially true in the sense that an infection may, like the other causes mentioned above, be the injury that starts the neuritis that is characteristic of the disease.

Zoster occurs at all ages. Lomer has reported a case occurring in an infant four days old; and examples of it in elderly individuals are not very rare in our clinics. It shows no predilection for the weak and debilitated; healthy and vigorous individuals are frequently attacked.

It occurs almost anywhere in the body, but is commonest in certain localities. My own experience would show it to be most frequent on the chest and the upper extremities; seven out of my thirty-two cases were in that locality. The patient before you is a good example of that localization known as zoster frontalis.

The symptomatology is so constant that it is hardly necessary to inquire into the anamnesis. For a varying number of days the patient suffers from burning, itching, and neuralgic pains in the area of skin that is about to be affected. Listlessness, anorexia, and the other symptoms of a moderate fever may be present. Examination reveals nothing locally, save perhaps a slight tenderness to deep pressure over the roots of the spinal nerves that supply the integument in question.

Suddenly there occurs an erythematous redness over a definite area of skin; and within a very few hours there appear on this area a group or groups of superficial vesicles. At first extremely minute, they are apparently papular; but before they become as large as the head of a pin, which occurs in a very few hours, the papules are topped with each a minute accumulation of serum, and have become vesicles. In thirty-six to forty-eight hours the vesicles have become pea-sized, and the serum has become milky or frankly purulent. By the third and fourth days adjacent and growing pustules have coalesced, and blebs of varying size have formed. If now they are not ruptured, the sero-pus is gradually absorbed, the bleb shrinks, and by the end of the week the affected area is covered with shriveled epidermis, under which repair of the destroyed area slowly takes place. If the pustules or blebs are ruptured, an excoriated or ulcerated surface is left behind, which heals in the course of the second or third week with the production of more or less new connective or scar tissue. Depressed cicatrices of varying size and depth are left behind; at first they are pigmented red or brown, but in the course of time they whiten out, like other neoplastic inflammatory tissue.

Whilst this first group or set of groups of vesicles, which marks the involvement in the inflammatory process of a bundle of nerve twigs, is going through these various stages, other bundles have become affected, and other groups of vesicles have appeared on other areas of skin, and even amongst the groups that appeared first. A few hours or several days may intervene between the birth of the successive groups or crops of the eruption; but each set appears in the same way and runs its own independent course, irrespective of the other older and younger groups around it. Thus at the same time we may see a group of red and pigmented scars representing the first efflorescence; excoriated surfaces with the semi-detached epithelium still covering them; groups of coalescent bullæ; assemblages of pustules and vesicles, large and small, and reddened areas where the primary erythema has but just appeared. Each successive crop runs its entire course independently. The other crops, even if the area occupied by them is intermingled, do not interfere with it. The malady advances "Schubweise," "a pouces successive," as our continental brethren say, as successive nerve twigs are involved in the inflammation.

The material that fills the vesicles and blebs is serum, sero-pus, or pus. In exceptional cases only is the inflammation severe enough to lead to the diapedesis of red as well as of white blood cells; in which case we have what is called zoster hemorrhagicus. Very rarely indeed is the inflammatory stasis of sufficient extent and duration to cause gangrene; giving us the zoster gangrenosus.

Unilaterality is a marked characteristic of the disease, as one would suppose from the nerve lesions that cause it. It is a superstition of the laity that if shingles becomes bilateral, if it encircles the body, the patient is doomed. This is not the case, and bilateral zoster, though rare, does occur. Almost invariably one attack protects against a recurrence of the disease, and we can promise our patients that they will not have it again. Nevertheless, there are exceptions to this rule also, and Hebra has recorded a case in which there were a dozen or more attacks in the course of several years.

The prognosis of zoster is good, save when the forehead and eyeball is affected. Suppurative panophthalmitis and destruction of the eyeball sometimes occurs in spite of all our efforts to save the organ. Obstinate neuralgias, that may plague the patient for years, may be left behind, but recovery is perfect, as a rule, when the disease ends.

The various cases are distinguished in accordance with their localization, and the one before you is a combination of Z. supra and infra-maxillaris, and Z. occipito-collaris. As you are aware, the inferior maxillary nerve, the third division of the fifth or trifacial, supplies the skin of the side of the head, the external ear, and the lower face. It resembles a spinal nerve in that it is composed of a motor and a sensory portion ; and the sensory filaments pass through the gasserian ganglion, which is analogous to the ordinary ganglion of a spinal nerve. Branches of the cervical plexus supply the skin of the neck and shoulder. In this case we may assume, then, that the gasserian ganglion of the inferior maxillary division of the fifth and the ganglia of the upper cervical nerves are the structures that are involved in the inflammation that is at the root of the disease.

I have comparatively little to say as regards treatment. If we had seen this patient at an earlier stage of the disease, when the pain and burning in the area to be affected were the prominent symptoms, it would have been proper to give him phenacetine or quinine in five to fifteen-grain doses three times daily. Arsenic in the shape of Fowler's Solution, thus:

℞ Sol. Arsen. Fowleri.
Aq., cinnamoni............ .............. ............. .... . .......... aa.ʒ i.
M. Sig. Six to twenty drops, after eating, in water.

Or solid:

℞ Acid. arseniosi........................................ .... ...........gr. ss.
Pulv. piper. nig....... .. .................. ..........................ʒ ss.
Extract. liquirit. q. s. ut ft. pil. No. XXX.
M. Sig. One two or three times daily, after eating, is indicated in all cases. Sometimes the pain is so severe that morphine, either by the mouth or hypodermically, must be employed.

As it is now, there is nothing to do but to protect and soothe the inflamed surfaces, and await the termination of the disease. A twenty per cent. mixture of chloroform and olive oil, or a five to twenty per cent. cocaine ointment will be found useful. In this case we will order the following, which is excellent for relieving the local inflammatory condition of the skin:

℞ Tr. opii.............. ................. ..... ............ ....... .........ʒ i.

Ac. carbolic............... .... .................. .... ... ...............gtt. iii.

Ol. amygd. dulc.............. ..... ........................... ......ʒ ii.

Adip. lanae............ ............ ............. ....................℥ ii.

M. Ft. ungt.

This mitigates the pain and the itching, keeps the surfaces moist and prevents their adhering to the clothes and the dressings, and promotes rapid and satisfactory healing.

Patients after an attack of zoster are frequently much debilitated, more especially from the loss of sleep that the accompanying neuralgia entails. When the two to three weeks of the disease is over, the bitter tonics, iron, cod liver oil, etc., are indicated.

37 *West 50th Street, New York City.*

## Figures and Tables

**Figure f1:**